# Anti-Inflammatory and Anti-Hyperuricemic Effects of Chrysin on a High Fructose Corn Syrup-Induced Hyperuricemia Rat Model via the Amelioration of Urate Transporters and Inhibition of NLRP3 Inflammasome Signaling Pathway

**DOI:** 10.3390/antiox10040564

**Published:** 2021-04-06

**Authors:** Yi-Hsien Chang, Yi-Fen Chiang, Hsin-Yuan Chen, Yun-Ju Huang, Kai-Lee Wang, Yong-Han Hong, Mohamed Ali, Tzong-Ming Shieh, Shih-Min Hsia

**Affiliations:** 1 Graduate Institute of Metabolism and Obesity Sciences, College of Nutrition, Taipei Medical University, Taipei 11031, Taiwan; peggy4103@gmail.com; 2 School of Nutrition and Health Sciences, College of Nutrition, Taipei Medical University, Taipei 11031, Taiwan; yvonne840828@gmail.com (Y.-F.C.); hsin246@gmail.com (H.-Y.C.); d04641004@ntu.edu.tw (Y.-J.H.); 3 Department of Nutrition, I-Shou University, Kaohsiung 84001, Taiwan; yonghan@isu.edu.tw; 4 Department of Nursing, Ching Kuo Institute of Management and Health, Keelung 20301, Taiwan; kellywang@tmu.edu.tw; 5 Clinical Pharmacy Department, Faculty of Pharmacy, Ain Shams University, Cairo 11566, Egypt; mohamed.aboouf@pharma.asu.edu.eg; 6 School of Dentistry, College of Dentistry, China Medical University, Taichung 40402, Taiwan; tmshieh@mail.cmu.edu.tw; 7 School of Food and Safety, Taipei Medical University, Taipei 11031, Taiwan; 8 Nutrition Research Center, Taipei Medical University Hospital, Taipei 11031, Taiwan

**Keywords:** chrysin, hyperuricemia, inflammasome, uric acid, gout

## Abstract

Hyperuricemia is the main cause of gout and involved in the occurrence of many other diseases such as hyperlipidemia and hypertension correlated with metabolic disorders. Chrysin is a flavonoid compound found naturally in honey, propolis, and mushrooms and has anti-inflammatory and antioxidant effects. However, its mechanism of action is not clear yet. This study investigated the mechanism of chrysin’s anti-hyperuricemic effect in hyperuricemia-induced rats fed with high-fructose corn syrup. Orally administrated chrysin for 28 consecutive days effectively decreased uric acid by inhibiting the activity of xanthine oxidase (XO) in the liver. Moreover, chrysin markedly downregulated the protein expression of uric acid transporter 1 (URAT1) and glucose transporter type 9 (GLUT9) and upregulated the protein expression of organic anion transporter 1 (OAT1) and human ATP-binding cassette subfamily G-2 (ABCG2). In addition, chrysin showed prominent anti-oxidative and inflammatory effects as the malondialdehyde (MDA) and interleukin 1 beta (IL-1β) concentration was reduced in both rat kidney and serum, which aligned with the inhibition of NOD-like receptor family pyrin domain containing 3 (NLRP3) inflammasome signaling pathway activation. Collectively, our results strongly suggest that chrysin exhibits potent anti-hyperuricemic and anti-inflammatory effects that may yield new adjuvant treatments for gout.

## 1. Introduction

Hyperuricemia occurs due to an imbalance in uric acid metabolism with accumulated serum uric acid (UA) that exceeds the saturation concentration (6.4–7.0 mg/dL) and results in many diseases including gout [1], chronic kidney disease (CKD) [2,3], cardiovascular disease [4], and metabolic syndrome [5]. According to the Nutrition and Health Surveys in Taiwan (NAHSIT) 2005–2008, the prevalence of hyperuricemia was higher in males (prevalence: 21.6%) than females (prevalence 9.57%), and there was a higher prevalence in 19–44.9-year-old males (prevalence 22.1%) than in the elderly (prevalence 10.1%) [6]. Briefly, UA is the final product of purine metabolism under the effect of the xanthine oxidase (XO) enzyme in the liver. About 70% of uric acid is excreted through the kidney and secreted in the urine, while the remaining 30% is excreted in the feces from the intestine [7]. The excretion of uric acid relies on the transport proteins in the proximal tubules of the kidney, whose function is to regulate the secretion of uric acid in the blood and filtrate. Uric acid-related transport proteins include uric acid reabsorption transporters (urate transporter protein-1, URAT1; glucose transporter-9, GLUT9) and uric acid excretion transporters (organic anion transporter, OAT1; human ATP-binding cassette subfamily G-2, ABCG2) [8]. Therefore, the balance of secretion and the reabsorption of uric acid is a critical factor that modulates uric acid concentration in serum.

The main cause of hyperuricemia is the intake of purine-rich foods such as meat, sugary drinks, and seafood. Results from the 2013–2016 Nutrition and Health Study in Taiwan (NAHSIT) showed that more than 80% of Taiwanese aged from 19 to 44 consumed more than one sugar-sweetened beverage per day [9]. These high-fructose-content beverages increase fructose metabolites, with subsequent consumption of a large amount of adenosine triphosphate, and an increase in the production of uric acid. This might explain the high prevalence of hyperuricemia among the Taiwanese [9]. In addition, fructose competes with uric acid to be excreted by the kidneys, reducing the rate of uric acid excretion and increasing the concentration of uric acid in the blood [10].

The increase of serum uric acid concentration accompanied with the excess production of reactive oxygen species (ROS) promoted the activation of inflammasomes [11]. Inflammasomes include NOD-like receptor family pyrin domain containing 3 (NLRP3), apoptosis-associated speck-like protein containing a caspase recruitment domain (ASC), and pro caspase-1, which activate cysteine-containing aspartate-specific protease-1 (caspase-1). Caspase-1 promotes the production of active inflammatory cytokine interleukin 1β (IL-1β), releases it outside the cell, promotes the occurrence of inflammation, and accelerates the process of kidney disease [12].

The first line treatment for hyperuricemia is allopurinol, which can inhibit xanthine oxidase activity and reduce uric acid production [13]. However, allopurinol has many side effects such as allergies, gastrointestinal discomfort, skin and mucous membrane necrosis, liver necrosis, and poor renal function [14]. Furthermore, long-term treatment could affect the lipid profile with increases in low density lipoprotein cholesterol (LDL-C), triglycerides (TG), and total cholesterol (TC), which in turn increase the atherogenic index and cause metabolic syndrome and a pronounced risk of heart diseases [15]. Considering the lower toxicity of natural compounds and the human body’s tolerance to them, an increasing number of studies have been conducted to explore the effects of various natural extracts in improving diseases [16,17].

Chrysin is a flavonoid with a 5,7-dihydroxyl structure and is found in honey, propolis, and mushrooms [18]. Previous studies have pointed out that chrysin has anti-cancer [19], anti-oxidant [20], anti-inflammatory [21], anti-anxiety [22], and liver-protective effects [23]. In addition, it was mentioned that, in an adenine-induced chronic kidney disease rat model, the intervention of chrysin had a protective effect on the kidneys by reducing the concentration of serum creatinine (CRE), blood urea nitrogen (BUN), and tumor necrosis factor alpha (TNF-α) [24]. The effect of chrysin on the regulation of uric acid and the improvement of kidney inflammation has not been fully explored. Therefore, in this study, we investigated the anti-hyperuricemic effects of chrysin as well as its mechanism including its regulation of urate-related transporter proteins and oxidative inflammasome activation.

## 2. Materials and Methods

### 2.1. Reagents

Allopurinol and carboxymethyl cellulose (CMC-Na) were purchased from Sigma-Aldrich (St. Louis, MO, USA). High-fructose corn syrup (90% fructose and 5% glucose) was purchased from Fonen and Fonher Enterprise Co. Ltd. (Tainan, Taiwan). Chrysin, a yellow powder and soluble in 0.5% CMC-Na solution, was purchased from Sigma-Aldrich (St. Louis, MO, USA), with a purity of ≥ 98%. The ELISA kit of xanthine oxidase (XO) was purchased from Cayman Chemical (Ann Arbor, MI, USA). The ELISA kit of IL-1β was purchased from BioLegend (San Diego, CA, USA). The rabbit polyclonal anti-ABCG2 and anti-OAT1 were obtained from Abcam (Cambridge, MA, USA). The rabbit polyclonal anti-GLUT9 was obtained from Millipore (Billerica, MA, USA), and anti-URAT1 was obtained from Proteintech (Rosemont, IL, USA). The mouse polyclonal anti-ASC and β-actin were obtained from Santa Cruz Biotechnology (Santa Cruz, CA, USA). The rabbit polyclonal anti-caspase-1 was obtained from Cell Signaling Technology (Danvers, MA, USA), and anti-NLRP3 was obtained from NOVUS Biologicals (Littleton, CO, USA).

### 2.2. Experimental Animal Model

Male Sprague-Dawley (SD) rats (six weeks old) were purchased from BioLASCO Taiwan Co. Ltd. (Taipei, Taiwan). The rats were raised in the Experimental Animal Center of Taipei Medical University (Taipei, Taiwan). The breeding environment was controlled at 25 ± 1 °C at a relative humidity of 60–70%, and at 12-h light and dark cycles. After one week of an adaptation period, 50 rats were randomly divided into six groups, C (Control group), the HFCS sham group (10% high-fructose corn syrup), CH50 (Chrysin 50 mg/kg body weight (bw)), CH100 (Chrysin 100 mg/kg bw), CH150 (Chrysin 150 mg/kg bw) [24], and AP (positive control group—allopurinol 10 mg/kg bw) [25]. All animal studies were conducted according to the protocols approved by the Institutional Animal Care and Use Committee (IACUC) of Taipei Medical University (Permit No.: LAC-2019-0265).

**Figure 1:**
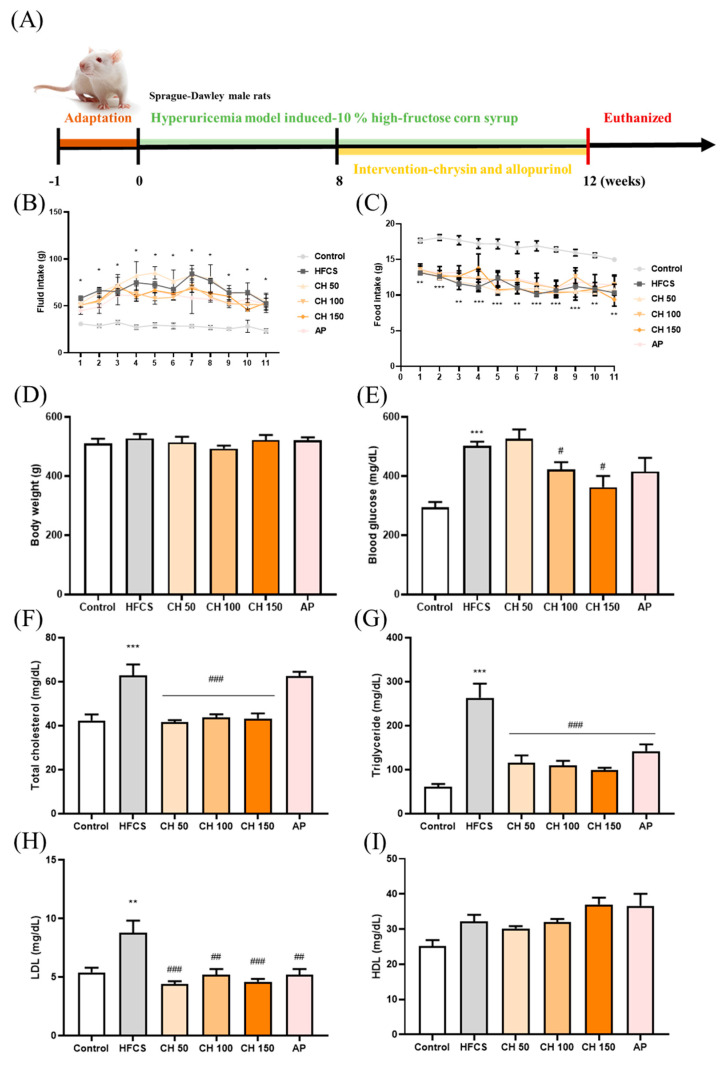
Effects of chrysin on food intake, fluid intake, body weight, serum glucose, and lipid profile. (**A**) Experimental flow chart, (**B**) fluid intake, (**C**) food intake, (**D**) body weight, (**E**) blood glucose, (**F**) total cholesterol, (**G**) triglycerides, (**H**) LDL, and (**I**) HDL level. C: control; HFCS: high-fructose corn syrup group (10% high-fructose corn syrup solution); AP: allopurinol (10 mg/kg BW); CH 50: chrysin (50 mg/kg bw); CH 100: chrysin (100 mg/kg bw); CH 150: chrysin (150 mg/kg bw). Data are represented as mean ± SEM. (*n* = 4–6 per group). * *p* < 0.05, ** *p* < 0.01, *** *p* < 0.001 versus the control group. ^#^ *p* < 0.05, ^##^ *p* < 0.01, ^###^ *p* < 0.001 versus the HFCS group.

After one week of the adaptation period, except for the control group, the other five groups were given a 10% high-fructose syrup solution for a total of 12 weeks (until the end of the study) to induce hyperuricemia. The intervention of chrysin (50, 100, and 150 mg/kg bw) or allopurinol (10 mg/kg bw) occurred from Week 8 to Week 12 (four weeks of treatment), and the intervention was given by oral gavage once daily, while the control group and HFCS sham group were administrated a CMC-Na solution, also by gavage. The intervention lasted for four weeks, and all rats were sacrificed at the 12th week of the experimental period. The general drinking water given to the control group or the 10% high-fructose syrup solution given to the other five groups were changed every 3–4 days, and water intake and food intake were measured. The kidney and liver were weighed, and the ratio of tissue weight and body weight were calculated to determine the value of tissue index (Supplementary Table S1). Figure 1A summarizes the experimental flow chart.

### 2.3. Sample Collection

The night before the sacrifice, the rats were placed in a metabolic cage to collect urine for over 4 h. After the supernatant was centrifuged at 4 °C and 3000× *g* for 10 min, it was collected in a 15 mL centrifuge tube and stored at −80 °C; blood samples were centrifuged at 4000× *g* for 10 min at 4 °C to obtain serum and stored at −80 °C for subsequent analysis.

### 2.4. Urine and Blood Biochemical Analysis

The serum and urine samples were analyzed by Taipei Medical University Hospital. Serum uric acid (UA), blood urea nitrogen (BUN), creatinine (CRE), blood glucose, total cholesterol (TC), triglyceride (TG), low-density lipoprotein cholesterol (LDL-C), high-density lipoprotein cholesterol (HDL-C), urine UA, and urine CRE concentration were detected using an automatic chemical analyzer (Roche Diagnostics, Rotkreuz, Switzerland).

### 2.5. Enzyme-Linked Immuno-Sorbent Assay (ELISA)

ELISA kits were used to measure the serum and kidney inflammatory factor index IL-1β (Biovision, K4796-100) and the Xanthine Oxidase Fluorometric Assay Kit (Cayman Chemical, Ann Arbor, MI, USA) in the liver. The manufacturer’s instruction was followed and a VERSA Max microplate reader was used for measurement (Molecular Devices, San Jose, CA, USA).

### 2.6. Lipid Peroxidation: Malondialdehyde Concentration

Plasma malondialdehyde (MDA) was measured using the “Thiobarbituric acid reactive substances (TBARS) Assay Kit” (Cayman Chemical, Ann Arbor, MI, USA) according to a previous study [26], and the manufacturer’s instructions. A VERSA Max microplate reader was used for measurement (Molecular Devices, San Jose, CA, USA).

### 2.7. Western Blot Analysis

Kidney tissues (about 0.1 g) were homogenized with a three-fold greater volume of lysis buffer, the homogenate was centrifuged at 12,000× *g* for 30 min at 4 °C, the supernatant was aspirated, and the protein concentration in the supernatant was determined using the BCA kit assays.

A volume containing 70–100 μg of protein samples was eluted with SDS-PAGE gel (10%) for colloidal electrophoresis, and the protein was then transferred to a PVDF membrane, followed by blocking by 5% skimmed milk powder at room temperature for 1 h. The membrane was then incubated with rabbit anti-GLUT9 (1:2000), rabbit anti-URAT1 (1:1000), rabbit anti-OAT1 (1:500), rabbit anti-ABCG2 (1:500), rabbit anti-NLPR3 (1:500), rabbit anti-caspase 1 (1:500), mouse anti-ASC (1:250), and mouse anti-β-actin (1:1000) at 4 °C overnight. The PVDF membrane was washed three times with TBST for 10 min each time. An appropriate secondary antibody (1:10,000, anti-rabbit IgG, or anti-mouse IgG) was added for 1 h at room temperature followed by washing three times, 10 min each, using TBST. The desired bands were visualized using a UVP Biospectrum AC System to capture the signal. The data were quantitatively analyzed using ImageJ software.

### 2.8. Statistical Analysis

The data were expressed as mean ± standard error of the mean (SEM) and GraphPad Prism version 7.0 (GraphPad, San Diego, CA, USA) was used for statistical analysis, and the statistical differences were tested using a Student’s t-test and a one-way analysis of variance (ANOVA) with a Mann–Whitney U test or the Tukey’s post-hoc test. A *p* < 0.05 was considered statistically significant.

## 3. Results

### 3.1. Effects of Chrysin on Food Intake, Fluid Intake, Body Weight, and Metabolic Markers in Hyperuricemic Rats

Following the treatment with either chrysin or allopurinol by oral gavage once daily for four weeks (Figure 1A), there was no change in tissue weight, tissue index, or kidney histology appearance (Supplementary Table S1 and Figure 1), indicating no apparent toxicity effect from chrysin treatment. Compared with the control group, the fluid intake was significantly increased in the HFCS group (Figure 1B), while the food intake was significantly decreased (Figure 1C); interestingly, no significant difference was observed in body weight (Figure 1D) among all groups. In addition, biochemical analyses showed that HFCS significantly increased serum glucose level and disrupted the serum lipid profile, where total cholesterol, triglycerides, and LDL increased compared with the control group. Interestingly, four weeks of chrysin intervention significantly improved these metabolic markers, as all aforementioned markers were significantly decreased (Figure 1E–I). HDL did not show significant changes in response to the treatments, possibly due to the variants between each other.

### 3.2. Effects of Chrysin on High-Fructose Corn Syrup Group (HFCS)-Induced Renal Dysfunction

**Figure 2:**
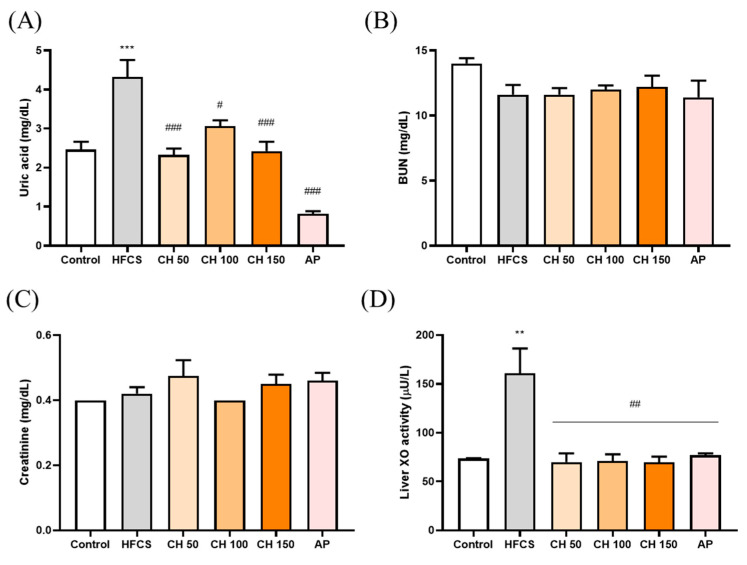
Effects of chrysin on (**A**) serum uric acid levels, (**B**) serum blood urea nitrogen (BUN), and (**C**) serum creatinine, (**D**) liver XO activity. C: control; HFCS: high-fructose corn syrup group (10% high-fructose corn syrup solution); AP: allopurinol (10 mg/kg bw); CH 50: chrysin (50 mg/kg bw); CH 100: chrysin (100 mg/kg bw); CH 150: chrysin (150 mg/kg bw). Data are represented as mean ± SEM (*n* = 4–6 per group). ** *p* < 0.01, *** *p* < 0.001 versus the control group. ^#^ *p* < 0.05, ^##^ *p* < 0.01, ^###^ *p* < 0.001 versus the HFCS group.

HFCS significantly elevated the serum level of uric acid after 12 weeks of treatment, while treatment with different doses of chrysin and allopurinol could significantly reverse the serum uric acid level back to a normal level (Figure 2A). However, neither HFCS nor chrysin showed any effects on kidney function indices such as blood urea nitrogen and creatinine (Figure 2B,C). We further examined the XO enzyme activity in the liver in response to different treatments. The results indicated that the HFCS group significantly increased the XO activity level (Figure 2D). Notably, treatment with chrysin opposed the HFCS-induced uric acid increase via the reduction of XO activity (Figure 2D).

### 3.3. Effects of Chrysin on Uric Acid Excretion in Hyperuricemic Rats

**Figure 3:**
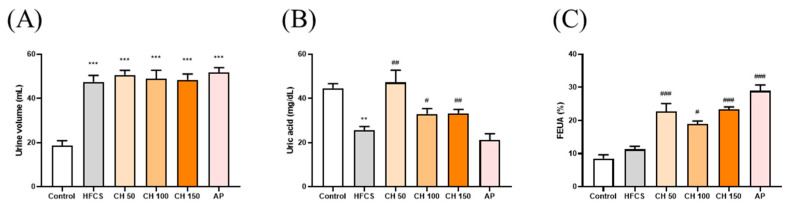
Effects of chrysin on (**A**) urine volume, (**B**) urine uric acid, and (**C**) fraction excretion of uric acid (FEUA). C: control; HFCS: high-fructose corn syrup group (10% high-fructose corn syrup solution); AP: allopurinol (10 mg/kg bw); CH 50: chrysin (50 mg/kg bw); CH 100: chrysin (100 mg/kg bw); CH 150: chrysin (150 mg/kg bw). Data are represented as mean ± SEM (*n* = 4–6 per group). ** *p* < 0.01, *** *p* < 0.001 versus the control group. ^#^ *p* < 0.05, ^##^ *p* < 0.01, ^###^ *p* < 0.001 versus the HFCS group. FEUA (%) values were calculated using the following formula: FEUA (%) = (Urine UA × serum CRE)/(Serum UA × Urine CRE) × 100.

The results showed that the urine volume in the HFCS group was significantly higher than the control group. However, there were no significant differences in the chrysin groups at doses of 50, 100, and 150 mg/kg bw compared with the HFCS group (Figure 3A). The urine uric acid concentration in the HFCS group was significantly lower than that in the control group (Figure 3B). Importantly, intervention with different doses of chrysin (50, 100, and 150 mg/kg) significantly increased the uric acid excretion in urine (Figure 3B). In addition, both the intervention of chrysin (50, 100, and 150 mg/kg bw) or allopurinol (10 mg/kg bw), compared with the HFCS group, significantly increased the fraction excretion of uric acid (FEUA), which reflects the reduction of serum uric acid in hyperuricemic rats (Figure 3C). Collectively, these results suggest that chrysin reduced the production of uric acid and increased urinary uric acid excretion by inhibiting the activity of XO.

### 3.4. Effects of Chrysin on the Expression of Urate-Related Transporter Proteins OAT1, ABCG2, URAT1, and GLUT9 in the Kidney of Hyperuricemic Rats

**Figure 4:**
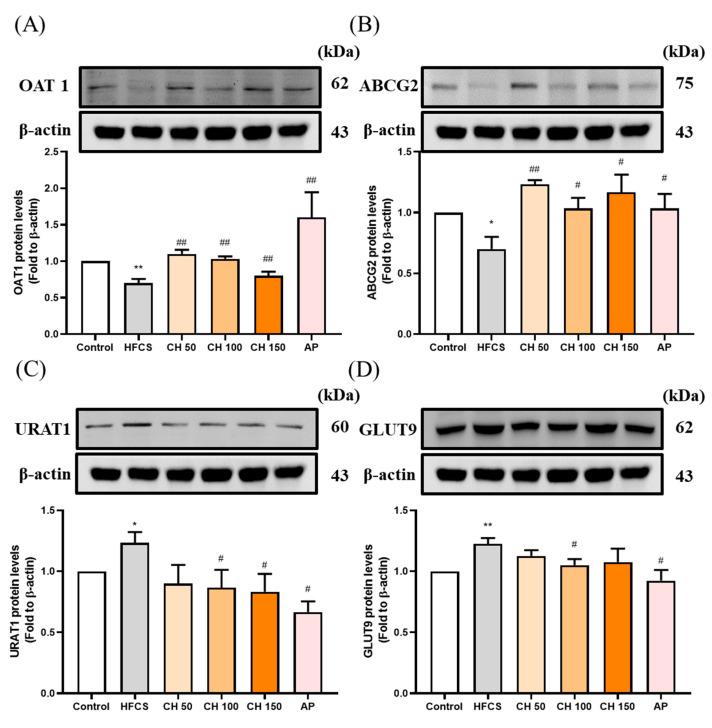
Effects of chrysin on urate-related transporter proteins expressions (**A**) OAT1, (**B**) ABCG2, (**C**) URAT1, and (**D**) GLUT9 in the kidney of hyperuricemic rats. C: control; HFCS: high-fructose corn syrup group (10% high-fructose corn syrup solution); AP: Allopurinol (10 mg/kg bw); CH 50: chrysin (50 mg/kg bw); CH 100: chrysin (100 mg/kg bw); CH 150: chrysin (150 mg/kg bw). Data are represented as mean ± SEM (*n* = 4–6 per group^).^ * *p* < 0.05, ** *p* < 0.01 versus the control group. ^#^ *p* < 0.05, ^##^ *p* < 0.01 versus the HFCS group.

Western blot data showed that the protein expression of uric acid excretion proteins OAT1 and ABCG2 were significantly decreased in the HFCS group compared to the control group (Figure 4A,B), while protein expression of the uric acid reabsorption proteins URAT1 and GLUT9 were significantly increased (Figure 4C,D). Importantly, chrysin or allopurinol treatment upregulated the uric acid transporters of OAT1 and ABCG2 and downregulated the uric acid reabsorption proteins of GLUT9 and URAT1, reversing the effects of HFCS (Figure 4A–D).

### 3.5. Effects of Chrysin on Pro-Inflammatory Cytokine IL-1β in Fructose-Induced Hyperuricemic Rats

**Figure 5:**
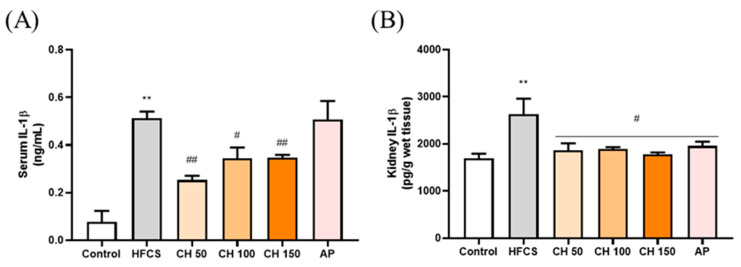
Effects of chrysin on pro-inflammatory cytokine (**A**) IL-1β in serum and (**B**) IL-1β in renal tissues of fructose-induced hyperuricemic rats. C: control; HFCS: high-fructose corn syrup group (10% high-fructose corn syrup solution); AP: Allopurinol (10 mg/kg bw); CH 50: chrysin (50 mg/kg bw); CH 100: chrysin (100 mg/kg bw); CH 150: chrysin (150 mg/kg bw). Data are represented as mean ± SEM (*n* = 4–6 per group). ** *p* < 0.01 versus the control group. ^#^ *p* < 0.05, ^##^ *p* < 0.01 versus the HFCS group.

ELISA results showed that the IL-1β concentration was significantly higher in both the serum and kidney of the HFCS group than that of the control group. In contrast, different doses of chrysin (50, 100, and 150 mg/kg) were able to significantly reduce the expression of IL-1β back in the serum and kidney of the rats. Interestingly, chrysin had a better anti-inflammatory effect than the AP group (Figure 5).

### 3.6. Effects of Chrysin on the Oxidative-Stress-Induced Inflammasome Activation

**Figure 6:**
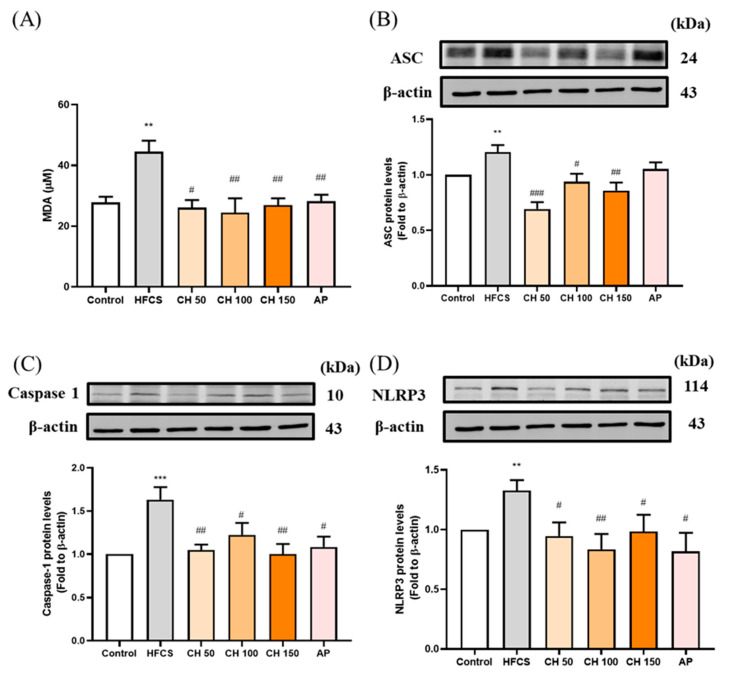
Effects of chrysin on oxidative inflammasome-activation (**A**) serum malondialdehyde (MDA) level and inflammasome protein expression of (**B**) ASC, (**C**) caspase-1, and (**D**) NLRP3 in the kidney of hyperuricemic rats. C: control; HFCS: high-fructose corn syrup group (10% high-fructose corn syrup solution); CH 50: chrysin (50 mg/kg bw); CH 100: chrysin (100 mg/kg bw); CH 150: chrysin (150 mg/kg bw). Data are represented as mean ± SEM (*n* = 4–6 per group). ** *p* < 0.01, *** *p* < 0.001 versus the control group. ^#^ *p* < 0.05, ^##^ *p* < 0.01, ^###^ *p* < 0.001 versus the HFCS group.

Since the urate-induced inflammasome pathway is activated in hyperuricemia through the production of reactive oxygen species (ROS). Chrysin treatments as well as allopurinol resulted in the reduction of MDA concentration, which increased in response to HFCS (Figure 6A). Moreover, HFCS significantly increased the protein expression of inflammasome-related proteins (ASC, pro-Caspase 1, NLRP3) (Figure 6B–D), while different doses of chrysin (50, 100, and 150 mg/kg) mitigated that effect and showed a significant reduction in all measured markers. These data showed that the increase in IL-1β concentrations in rat serum and kidney were activated by urate-induced inflammasome in the HFCS group, while chrysin reduced the oxidative stress and thus the activation of inflammasome, which in turn effectively reduced the concentration of the inflammatory factor IL-1β.

## 4. Discussion

Xanthine oxidase (XO) is an important enzyme that converts xanthine and hypoxanthine into uric acid, so a higher rate of activity of XO leads to the excessive production of uric acid [27]. In previous studies, it was shown that the C5 and C7 positions in the flavonoid structure are connected with hydroxyl groups, and C2 and C3 are connected by double bonds, which contribute to their inhibitory effect of XO activity [28]. Knowing that chrysin is a flavonoid with a 5,7-dihydroxyl structure, the results of this study confirmed that chrysin could significantly inhibit the activity of XO in the liver, thereby reducing the production of uric acid.

A hyperuricemia animal model can be classified in two ways: genetically modified and environmental-factor-induced [29]. Genetically induced models include uricase, GLUT9, ABCG2, and URAT1 knockout animal models. The disturbance of the uric acid metabolism can cause increased serum urate concentration, inflammation, and progressive kidney failure [30]. Environmentally induced animal models involve the chemical inhibition of uricase. Using an uricase inhibitor, potassium oxonate, to block the hepatic uricase can increase the serum uric acid level with urate nephropathy [31]. Simulating the dietary consumption of the progression in hyperuricemia involves a Western diet, a fructose diet, and a high fat diet [29]. A metabolic disorder and an increase in inflammation can induce hyperuricemia and nephropathy [32].

Excessive fructose intake is an important contributor to hyperuricemia, especially high-fructose syrup [33]. In previous studies, rats fed with 60% high-fructose [34] or a 10% high-fructose solution [35,36] were able to increase serum uric acid concentration and disrupt the lipid profile. According to the results of the current study, rats fed with a 10% high-fructose syrup solution for 12 weeks first induced a hyperuricemia pattern, in accordance with previous literature, with significantly increased serum uric acid, total cholesterol, triglycerides, and low-density lipid protein cholesterol concentration. Notably, the intervention of chrysin was able to significantly reduce uric acid concentration and improve dyslipidemia.

Creatinine and urea nitrogen are indicators of abnormal renal function, reflecting the ability of the kidneys to excrete protein metabolites. In previous studies, 150 mg/kg adenine and 250 mg/kg ethambutol induced hyperuricemia and increased serum uric acid concentration, blood urea nitrogen, and creatinine, with displayed renal dysfunction [37]. In the current study, a 10% high-fructose syrup solution induced hyperuricemia, but was unable to alter these renal function indexes. In the meantime, the intervention of chrysin was able to reverse the increased uric acid level without affecting renal function.

The related body of literature shows that a high fructose intake can reduce the ability of the kidneys to excrete uric acid, increase oxidative stress, and activate the inflammasome [38,39]. Fructose in metabolism can increase the UA production from amino acids, and long-term consumption can further decrease the UA excretion ability in kidneys [40], which increases serum uric acid levels. A UA production increase can increase the ROS level and alter XO activity [41]. UA production requires an energy production drive from the NAD^+^ accompany with the utilization of oxygen and generates superoxide and hydrogen peroxide [42]. Our results are consistent with previous studies. Rats were fed with a 10% high-fructose solution for 12 weeks, and urine uric acid concentration was significantly reduced in the control group. Moreover, our results indicate for the first time that the intervention of chrysin can improve the ability of the kidneys to excrete uric acid, counteracting the effect of high fructose intake, and eventually promote urinary uric acid excretion.

**Figure 7:**
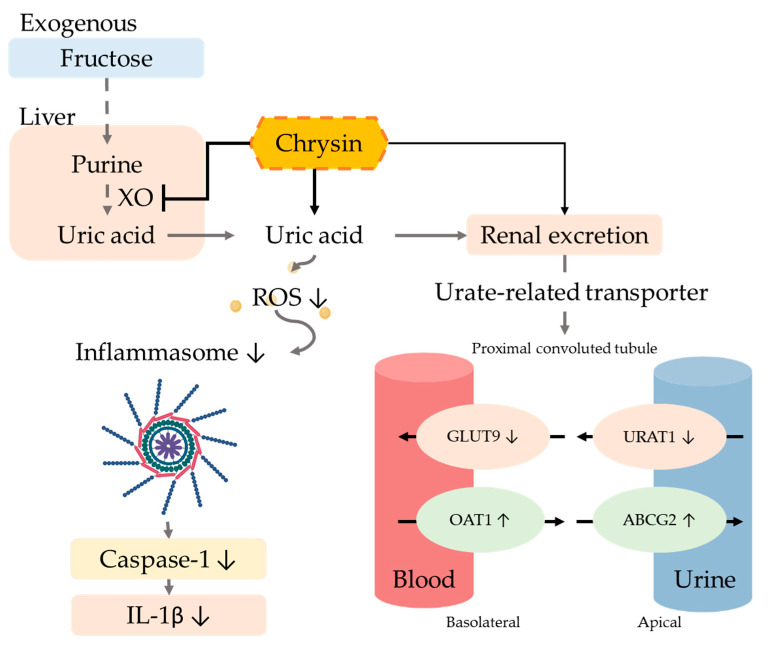
Potential mechanism of chrysin in hyperuricemic rats. The intervention of chrysin can effectively inhibit xanthine oxidase (XO) activity in the liver, thereby reducing the production of uric acid. Chrysin also reduces the reabsorption of uric acid by regulating uric acid-related transporters and promotes the excretion of uric acid in the urine, thus improving hyperuricemia. Finally, chrysin can reduce the IL-1β concentration by the reduction of oxidative stress and the inactivation of inflammasome.

The excretion of uric acid in the urine is mainly dependent on uric acid transport-related proteins in either the apical membrane or basement membrane of renal proximal tubule epithelial cells. Uric acid reabsorption transporters URAT-1 and GLUT9 are responsible for regulating the reabsorption of uric acid and thus control the content of uric acid return back into the blood. On the other hand, the uric acid excretion transporters ABCG2 and OAT1 are responsible for regulating uric acid excretion via secreting uric acid into the urethra [43]. Previous studies showed that six weeks of treatment of a 10% fructose diet altered the urate transporter regulation and increased the uric acid serum level [44]. Moreover, many natural extracts have been explored for efficacy using animal models of hyperuricemia such as quercetin [36], resveratrol [45], fucoxanthin [46], green tea polyphenols [47], and longan extract [48], which can inhibit the reabsorption of uric acid by regulating uric acid-related transporters. The xanthine oxidoreductase (XOR) inhibitor can alter not only the transporter URAT1 and GLUT9 and decrease the purine concentration, but can further decrease the ROS level and prevent ROS-related cell injury [49]. A hypertension model indicated that the increase in ROS can influence the ROS-related signal transduction, alter the transporter ion modulation, and further change the uric excretion and reabsorption [50]. Our results demonstrated that using chrysin not only downregulated the expression of reabsorption proteins GLUT9 and URAT1, but also upregulated uric acid excretion proteins OAT1 and ABCG2 (Figure 7).

Hyperuricemia is known to accelerate the progression of kidney disease, and high concentrations of uric acid increase oxidative stress and promote inflammation progression [51]. Previous studies have pointed out that stimulating human primary renal tubular epithelial cells with high concentrations of uric acid increased the expression of the pro-inflammatory cytokine IL-1β, along with the activation of inflammasome proteins [52]. Considering chrysin’s natural antioxidant effects, it showed a hepatoprotective effect in a d-galactosamine-induced hepatitis study through the reduction of lipid peroxidation products [53]. Furthermore, in a knee osteoarthritis study, chrysin displayed its capability of inhibiting inflammasome [54]. In the current study, chrysin could effectively decrease IL-1β levels by the reduction of oxidative stress and the inactivation of inflammasome.

## 5. Conclusions

In summary, the results imply that chrysin exerts its hyperuricemia effect through antioxidant activity and the inactivation of inflammasome, subsequently improving conditions of hyperuricemia-related metabolic disease.

## Data Availability

The data presented in this study are available on request from the corresponding author.

## Supplementary Materials

The following are available online at https://www.mdpi.com/article/10.3390/antiox10040564/s1. Table S1: Effects of chrysin on tissue weight and tissue index on fructose induced hyperuricemic rats. Figure S1: Effects of chrysin on kidney histology in HFCS-induced hyperuricemic rats.

## Abbreviations

ABCG2	ATP-binding cassette subfamily G-2
ASC	Apoptosis-associated speck-like protein containing a caspase recruitment domain
BUN	Blood urea nitrogen
CRE	Creatinine
Caspase 1	Cysteine-containing aspartate-specific protease-1
FEUA	Fraction excretion of uric acid
GLUT9	Glucose transporter type 9
HDL-C	High density lipoprotein cholesterol
HFCS	High-fructose corn syrup
IL-1β	Interleukin 1 beta
LDL-C	Low density lipoprotein cholesterol
NLRP3	NOD-like receptor family pyrin domain containing 3
MDA	Malondialdehyde
OAT1	Organic anion transporter1
ROS	Reactive oxygen species
TC	Total cholesterol
TG	Triglyceride
UA	Uric acid
URAT1	Uric acid transporters1
XO	Xanthine oxidase
