# Supplementary material for: Anti-Inflammatory and Anti-Hyperuricemic Effects of Chrysin on a High Fructose Corn Syrup-Induced Hyperuricemia Rat Model via the Amelioration of Urate Transporters and Inhibition of NLRP3 Inflammasome Signaling Pathway
Source: Antioxidants (Basel). 2021 Apr 6;10(4):564. doi: 10.3390/antiox10040564 (PMC8067405; doi:10.3390/antiox10040564)
Supplement: Supplementary file 1 [file antioxidants-10-00564-s001.pdf]

Anti-Inflammatory and Anti-Hyperuricemia Effects of Chrysin on High Fructose Corn Syrup-Induced Hyperuricemia Rats via Ameliorates of Urate Transporters and the Inhibition of NLRP3 Inflammasome Signaling Pathway

Yi-Hsien Chang[1], Yi-Fen Chiang[2†], Hsin-Yuan Chen[2,4], Yun-Ju Huang[2], Kai-Lee Wang[3], Yong-Han Hong[4], Mohamed Ali[5], Tzong-Ming Shieh[6], Shih-Min Hsia [1,2,6,7*]

[1] Graduate Institute of Metabolism and Obesity Sciences, College of Nutrition, Taipei Medical University, Taipei, Taiwan; peggy4103@gmail.com (Y.-S.C.)
[2] School of Nutrition and Health Sciences, College of Nutrition, Taipei Medical University, Taipei 11031, Taiwan; yvonne840828@gmail.com (Y.-F.C.); hsin246@gmail.com (H.-Y.C.); d04641004@ntu.edu.tw (Y.-J.H.);
[3] Department of Nursing, Ching Kuo Institute of Management and Health, Keelung 20301, Taiwan; kellywang@tmu.edu.tw
[4] Department of Nutrition, I-Shou University, Kaohsiung 84001, Taiwan; hsin246@gmail.com (H.-Y.C.); yonghan@isu.edu.tw (Y.-H. H)
[5] Clinical Pharmacy Department, Faculty of Pharmacy, Ain Shams University, 11566 Cairo, Egypt; mohamed.aboouf@pharma.asu.edu.eg (M.A.)
[6] School of Dentistry, College of Dentistry, China Medical University, Taichung 40402, Taiwan.; tmshieh@mail.cmu.edu.tw (T.-M.S.)
[7] School of Food and Safety, Taipei Medical University, Taipei 11031, Taiwan
[8] Nutrition Research Center, Taipei Medical University Hospital, Taipei 11031, Taiwan
* Correspondence: bryanhsia@tmu.edu.tw (S.-M.H.); Tel.: +886-273-61661-6558
† Equal contribution.

Supplementary Table 1.

Effects of chrysin on tissue weights, and tissue index in HFCS induced hyperuricemic rats

Group	Kidney weight(g)	Kidney index(%)	Liver weight(g)	Liver index(%)
Control	4.18±0.56	0.82±0.08	15.67±2.42	3.05±0.26
HFCS	4.27±0.40	0.81±0.06	19.22±3.73	3.62±0.45
CH 50	4.58±0.47	0.91±0.06	18.13±1.84	3.54±0.34
CH 100	4.31±0.32	0.90±0.09	17.34±1.62	3.52±0.29
CH 150	4.25±0.21	0.87±0.06	17.52±2.84	3.34±0.26
AP	4.71±0.34	0.82±0.05	18.40±2.47	3.53±0.41

Tissue index (%) = (tissue weight / body weight) × 100
Data are means ± S.E.M. (n=8-9 per group). HFCS, high-fructose corn syrup group (10 % high-fructose corn syrup solution); AP, positive control group (Allopurinol: 10 mg/kg BW); CH 50: chrysin (50 mg/kg BW); CH 100: chrysin (100 mg/kg BW); CH 150: chrysin (150 mg/kg BW).

Supplementary Figure 1.

Effects of chrysin on kidney histology in HFCS induced hyperuricemic rats

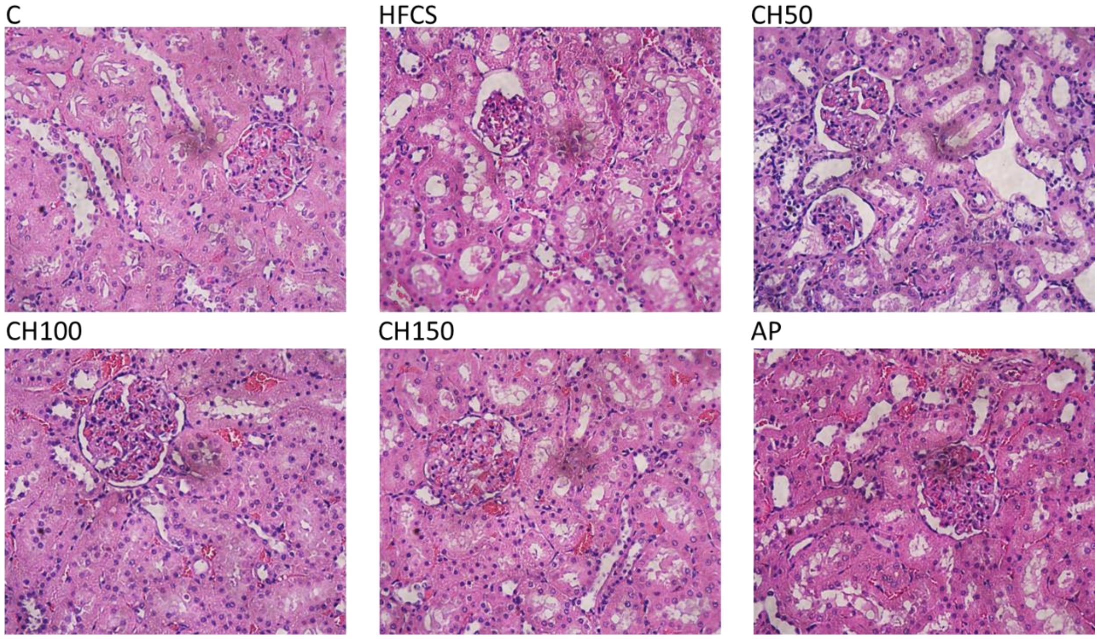

Representative images of kidney H&E staining.
Data are means ± S.E.M. (n=8-9 per group). HFCS, high-fructose corn syrup group (10 % high-fructose corn syrup solution); AP, positive control group (Allopurinol: 10 mg/kg BW); CH 50: chrysin (50 mg/kg BW); CH 100: chrysin (100 mg/kg BW); CH 150: chrysin (150 mg/kg BW).
